# Thromboelastography and clinical outcomes in peripheral arterial disease: a systematic review and narrative synthesis

**DOI:** 10.1308/rcsann.2025.0091

**Published:** 2026-01-20

**Authors:** J-A Broomfield, A Abidia, JP Gopal

**Affiliations:** ^1^ The Princess Alexandra Hospital NHS Trust, UK; ^2^ King's College Hospital NHS Foundation Trust, UK; ^3^ Cambridge University Hospital NHS Foundation Trust, UK

**Keywords:** Thromboelastography, Peripheral arterial disease, Thrombosis, Systematic review, Viscoelastic assay, TEG with platelet mapping

## Abstract

**Introduction:**

Thromboelastography (TEG) is a point-of-care test that provides a quantitative of measure of the dynamic changes in clot strength and viscoelastic properties of a whole blood sample. Although conventional coagulation tests are well established in vascular surgery, they do not identify the hypercoagulable state and response to antiplatelet therapy. The role of TEG in peripheral arterial disease (PAD) is unclear and its application as demonstrated in the literature has undergone limited appraisal. The objectives of our study were to identify whether TEG can inform individualised thromboprophylaxis and predict thrombotic events following re-vascularisation in PAD.

**Methods:**

We adhered to the Preferred Reporting Items for Systematic Reviews and Meta-Analyses (PRISMA) guidelines and a PRISMA checklist was completed. PubMed and Embase databases were searched from inception until October 2024 using the relevant Medical Subject Headings terms. Only full-text articles published in the English language reporting the outcomes in PAD with TEG or TEG with platelet mapping (TEG-PM) were analysed. The protocol was registered on the PROSPERO database (ID:CRD42024580627).

**Findings:**

The analysis included 14 studies. TEG-PM was able to quantify the response to antiplatelet therapy and potentially guide individualised thromboprophylaxis. The parameters maximum amplitude, platelet aggregation and platelet inhibition were able to predict thrombotic events. However, substantial heterogeneity in thromboprophylaxis, surgical procedures and comorbidities was observed in the studies.

**Conclusions:**

TEG-PM could serve as a valuable tool for tailoring antiplatelet therapy and predicting outcomes in patients with PAD. Further studies including randomised controlled trials are needed to validate the findings.

## Introduction

Thromboelastography (TEG) is a point-of-care test that provides a quantitative of measure of the dynamic changes in clot strength and viscoelastic properties of a whole blood sample. It differs from conventional coagulation tests in that it provides a more comprehensive measure of coagulation over time, including clot formation, strength and lysis.^1,2^ Although conventional coagulation tests are well established in vascular surgery, they have limitations.^2,3^ Platelet function testing provides crucial information regarding antiplatelet resistance but there is no consensus on its use in peripheral arterial disease (PAD).^4^ Among platelet function tests, TEG with platelet mapping (TEG-PM) is advantageous because it is a point-of-care test and also provides information about coagulation. TEG has been successfully implemented across different specialties.^5^ However, the role of TEG specifically in PAD has undergone limited appraisal and new evidence has emerged since the previous review.^6^

In PAD, conventional practices involve the use of anticoagulants or antiplatelet agents for maintaining graft patency and preventing thrombotic events in the perioperative phase and in the long term.^7,8,9^ Anticoagulant monitoring is implemented to ensure therapeutic efficacy and gauge bleeding risk when using vitamin K antagonists or unfractionated heparin.^1,10,11^ However, there is currently no agreed set standard for monitoring antiplatelet therapy or direct-acting oral anticoagulants (DOAC) such as factor Xa inhibitors.^1,12^ The utilisation of TEG in PAD has undergone limited appraisal but could serve as a valuable method for tailoring antiplatelet therapy and predicting patient outcomes**.**

There are various modifications of TEG via the addition of different clotting activators. Standard TEG, in which kaolin is used as an activator, can generate results that reflect activated partial thromboplastin time, the intrinsic pathway of the clotting cascade. Rapid TEG is a modification in which both tissue factor and kaolin are added. This generates more rapid results, by propagating both the intrinsic and extrinsic pathways of the clotting cascade. Heparinase TEG, via addition of heparinase, enables the detection of heparin. Functional fibrinogen TEG assesses the fibrin and platelet contribution to clot strength, and is achieved by the addition of a glycoprotein IIb/IIIa platelet inhibitor. TEG-PM is a further modification by addition of adenosine diphosphate (ADP) or arachidonic acid (AA). TEG-PM enables assessment of the inhibitory effect of antiplatelet agents (such aspirin or clopidogrel) on platelet function.^2^

**Figure 1:**
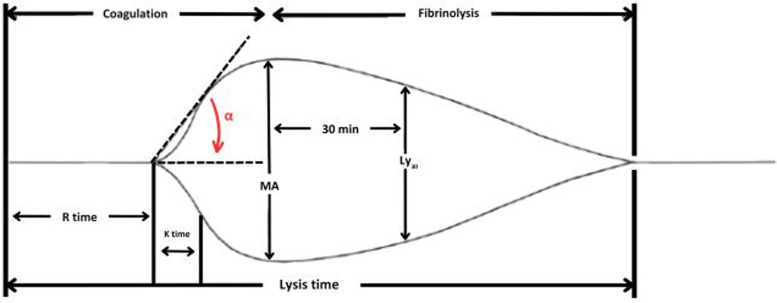
Example thromboelastogram

When assessing the generated thromboelastogram (Figure 1), known values correlate to different stages of clot formation. More detailed information regarding these parameters and their interpretation are given in Table 1. Each of these values is influenced by different factors, such as the concentration of coagulation factors, platelet volume, platelet function, factor deficiencies and the use of anticoagulation or antiplatelet agents.^5^

**Table 1:** Thromboelastogram parameters ^1,2,4^

Parameter	Graphical interpretation	Aspect of clot formation, propagation, strength and lysis	Clinical interpretation
R-time	Reaction time: measure of time from activation of the coagulation cascade to point of clot formation	• Activation of coagulation • Thrombin generation	Prolongation suggests anticoagulant usage or qualitative or quantitative deficiency of coagulation factors
K-time	Kinetic time: measure of time from the end of R-time, to when clot strength is 20mm	• Fibrin activation • Fibrin polymerisation	Prolongation suggests quantitative/qualitative fibrinogen or platelet deficiency
α-Angle	Alpha angle (α): the angle formed between the *x*-axis and the slope between R-time and K-time	• Fibrin activation • Fibrin polymerisation	Reduced α-angle suggests quantitative/qualitative fibrinogen or platelet deficiency
MA	Maximum amplitude: peak measure of clot strength attained, measured from the *x*-axis to the peak point of the curve	• Fibrinogen contribution to clot strength • Platelet contribution to the strength of the clot	Decreased MA suggests platelet deficiency, antiplatelet therapy usage, improper coagulation or factor/fibrin formation
Ly30	Lysis at 30min: percentage decrease in maximum clot strength (MA) at 30min	• Fibrinolysis	Increased Ly30 suggests state of hyperfibrinolysis, indicative of physiological states precipitating this; i.e. postoperative

Conventional coagulation tests include, but are not limited to: activated partial thromboplastin time, prothrombin time, international normalised ratio, activated clotting time and platelet count.^10,11^ Unlike TEG, conventional coagulation tests fail to reflect the dynamic interplay of the clotting cascade and platelets in whole blood, and are therefore are less illustrative physiologically and unable to comprehensively quantify an individual’s response to antiplatelet agents.^10^ Studies have recognised that across different patient populations, uniform responses to antiplatelet agents are not observed, particularly in differing responses in each sex.^13^^–^^15^ In certain patient populations, resistance to clopidogrel and aspirin has also been recognised, which encourages further evaluation of monitoring approaches.^16,17^

We present a systematic review of the current available evidence on the application of TEG in PAD, with a focus on TEG informing individualised treatment strategies and predicting thrombotic events following re-vascularisation**.**

## Methods

In conducting this systematic review, we adhered to the Preferred Reporting Items for Systematic Reviews and Meta-Analyses (PRISMA) guidelines and the PRISMA checklist was completed.^18^ PubMed and Embase databases were searched from inception until October 2024 using the following Medical Subject Headings (MeSH): “thromboelastography in peripheral vascular disease”, “thromboelastography in peripheral arterial disease”, “TEG and peripheral arterial disease”, “TEG and peripheral vascular disease”, “thromboelastography and lower limb re-vascularisation”, “TEG and lower limb re-vascularisation”, “thromboelastography and predicting thrombotic events”, “TEG and predicting thrombotic events’, **‘**thromboelastography and amputation” and “TEG and amputation”. To further refine searches when prompted, the following MeSH subheading terms were included: peripheral vascular disease, peripheral arterial disease and thromboelastography.

Articles were screened for relevancy by title, abstract and/or full article. Preclinical studies, review articles, conference abstracts, editorials, journal notes, letters to editors and animal studies were excluded. If articles were relevant but not available in the English language or full text, they were also excluded. The included articles examined patient populations aged over 18 years with PAD, who had undergone or were due to undergo peripheral re-vascularisation (either endovascular, open surgical or hybrid)**.**

Two independent reviewers screened all records and disagreements were resolved by a third reviewer. Articles that met the study inclusion criteria were assessed for quality and risk of bias using the Newcastle–Ottawa Scale (NOS) for non-randomised studies. Assessment was made at study (cohort, exposure) and outcome (assessment of outcome, follow-up) level. Included studies were scored on eight variables and the maximum score was 9 points (2 for comparability of cohorts and 1 each for the rest of the variable). Studies with a score of 7 or more were considered good-quality articles. Article quality was assessed by two independent reviewers and disagreements were resolved by a third reviewer. There was no minimum number of studies or duration of follow-up set for the data synthesis.

The following information was extracted: year of the study, author, study design, number of patients included, type of point-of-care test and timing of use, subtype of PAD, thromboprophylaxis regimen, type of re-vascularisation and outcome including bleeding. We grouped the studies into the following groups based on our objectives: (1) TEG/TEG-PM utility and ability to personalise thromboprophylaxis; and (2) TEG/TEG-PM in predicting thrombosis. The data and assessment are presented as tables and charts. No meta-analysis was performed because of heterogeneity in the end point and measures that precluded pooling of results. The protocol was prospectively registered on the PROSPERO database (ID: CRD42024580627).

## Findings

### Identification of studies

**Figure 2:**
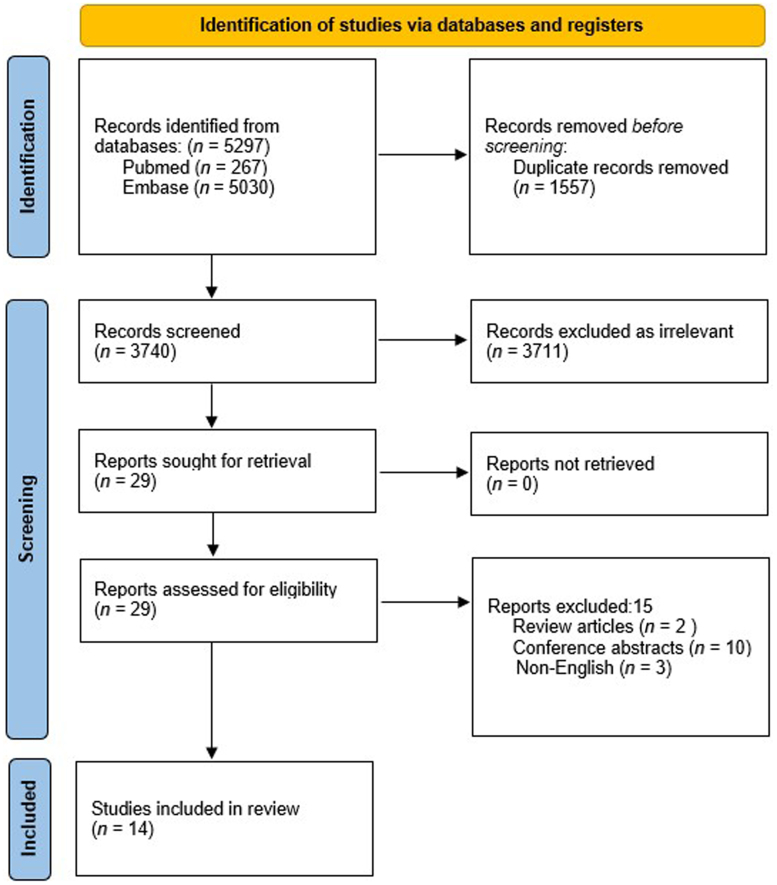
PRISMA flowchart

The initial search across 2 databases returned 5,297 articles (PubMed 267; Embase 5,030). After initial screening, 1,557 duplicate records were removed and 3,740 records were included for further screening, 3,711 of which did not meet the study criteria and were excluded. Twenty-nine records were sorted for retrieval and further assessed for eligibility. A further of 15 records were excluded (2 review articles, 10 conference abstracts and 3 records not in English). Finally, 14 studies met the study criteria and were included in the review. This is illustrated in the PRISMA flowchart (Figure 2).

### Study characteristics and quality assessment

All the studies included in this systematic review were single institutional studies from North America and Europe. The study period range between 2020 and 2023. The follow-up period varied between intraoperative period to 1-year post intervention. There were 2 case-control studies and 12 cohort studies. The included studies correspond to level 2b and level 3b on the Oxford CEBM 2011 hierarchy.^19^ The main study characteristics are illustrated in Table 2 and Table 3. Studies scoring 7 or greater on the NOS scale were regarded as good-quality studies. The study quality assessment is illustrated in Table 4. Among the included studies, 12 were of high quality and 2 were of low quality.

**Table 2:** Main study characteristics

Author (year)	Study design	Study period	Number of patients included (*n*)	Type of point-of-care testing	Timing of point-of-care testing	Outcome studied
Suarez Ferreira *et al* (2023)^20^	Cohort study^a^ Prospective	2020–2022	72	TEG-PM	Pre-intervention Post-intervention (1, 3 and 6 months)	• Platelet inhibition (clopidogrel + atorvastatin vs clopidogrel alone)
Hall *et al* (2023)^21^	Cohort study^a^ Prospective for PAD and retrospective for CAD	2020–2022	114	TEG-PM	Pre-intervention Post-intervention (daily for up to 5 days postoperatively if inpatient, 1, 3 and 6 months)	• Compare TEG-PM metrics between those with significant CAD and PAD and those with PAD alone, as well the incidence of MALE • Compare TEG-PM metrics between those who experienced MALE and those who did not along, as well as other thrombotic events such as DVT/PE, myocardial infarction, stroke and death
Hall *et al* (2023)^22^	Cohort study^a^ Prospective	2020–2022	141	TEG and TEG-PM	Pre-intervention Post-intervention (daily for up to 5 days postoperatively if inpatient, 1, 3 and 6 months	• Compare TEG-PM metrics between DOAC vs non-DOAC • Incidence of MALE, the associated antithrombotic regimen and TEG-PM profile
Lee *et al* (2024)^23^	Cohort study^a^ Prospective	2020–2023	64	TEG-PM	Pre-intervention (within 48h) Post-intervention (daily for up to 5 days postoperatively if inpatient, 1, 3 and 6 months)	• Compare occurrence of by-pass/in-stent stenosis; wound complications; bleeding events (in patients who transitioned from MAPT to DAPT • Influence of sex on efficacy of MAPT and DAPT • Compare platelet reactivity between open and endovascular procedures to elucidate the impact of the procedure on response to DAPT • Longitudinal comparison of antiplatelet reactivity
Majumdar *et al* (2023)^24^	Cohort study^a^ Prospective	2020–2022	162	TEG-PM	Pre-intervention (within 24h) Post-intervention (daily for up to 5 days postoperatively if inpatient, first outpatient visit, 3 and 6 months)	• Composite outcome of graft/stent thrombosis (radiological evidence of graft/stent failure; re-intervention to re-establish arterial flow; major limb amputation)
Martin *et al* (1994)^25^	Case-control^a^	Not mentioned	15	TEG	Intraoperative (5min prior to heparin administration, 5min after arterial occlusion, at 30-min intervals until anastomosis was complete)	• Assess whether empirical dose of heparin provides adequate anticoagulation when arterial clamping • Provide objective measure of intraoperative monitoring of heparin anticoagulation • Establish whether variability of heparin metabolism necessitates alternative approach to intraoperative anticoagulation
Suarez *et al* (2024)^26^	Cohort study^a^ Prospective	2020–2023	181	TEG and TEG-PM	Pre-intervention Post-intervention (1, 3 and 6 months) ACE protocol (only patients on antiplatelet therapy and undergoing endovascular re-vascularisation): TEG-PM at baseline, 1 week, 1, 2, 3 and 6 months post intervention	• Objectively quantify the impact of sex on platelet function in PAD patients aged >60 years taking antiplatelet and anticoagulant medications • Develop and test and personalised algorithm for thromboprophylaxis by incorporating platelet function testing
Suarez Ferreira *et al* (2024)^27^	Cohort study^a^ Prospective	2020–2023	149	TEG-PM	Pre-intervention Post-intervention (1, 3 and 6 months)	• Delineate sex-specific difference in platelet function and response to antiplatelet therapy in PAD patients on aspirin monotherapy
Suarez Ferreira *et al* (2024)^28^	Cohort study^a^ Prospective	2021–2023	158	TEG-PM	Pre-intervention Post-intervention (1, 3, 6 and 9 months)	• Prediction of increased risk of thrombosis using objective measures of clot strength
Shankar *et al* (2006)^29^	Case-control study^a^	Not defined	30	TEG	Aortic blood samples immediately prior and within 2min after last dose of iohexol prior to any intervention procedure	• Define the changes in coagulation adjacent to the site of contrast injection/potential angioplasty
Hall *et al* (2024)^30^	Cohort study^a^ Prospective	2020–2023	202	TEG-PM	Pre-intervention Post-intervention (daily for up to 5 days postoperatively if inpatient, 1, 3 and 6 months)	• Compare preoperative TEG-PM metrics between normal BMI and BMI>25 patients; and also between diabetic and non-diabetic patients • Compare TEG-PM metrics between thrombosis and no thrombosis group
Majumdar *et al* (2022)^31^	Cohort study^a^ Prospective	2020–2022	102	TEG-PM	Pre-intervention Post-intervention (daily for up to 5 days postoperatively if inpatient, first visit, 3 and 6 months)	• The need for operative intervention for surgical site infection/dehiscence • Incidence of non-surgical site infection (new ulcerative wound) in the index limb
Majumdar *et al* (2023)^32^	Cohort study^a^ Prospective	2020–2022	143	TEG-PM	Pre-intervention Post-intervention (daily for up to 5 days postoperatively if inpatient, 1, 3 and 6 months) Only pre-intervention sample was collected in those who failed re-vascularisation	• Comparison of platelet aggregation and platelet inhibition between MAPT vs DAPT (overall and at different time points: preoperative, postoperative inpatient, postoperative outpatient)
Lee *et al* (2024)^33^	Cohort study^a^ Prospective	2020–2023	228	TEG-PM	Pre-intervention Post-intervention (between 4 and 36h, 1, 3 and 6 months)	• Compare TEG-PM parameters between diabetic and non-diabetic patients (overall and at different time points) • Compare TEG-PM parameters within diabetic patients stratified based on HbA_1c_ levels • Compare the incidence of thrombotic events between diabetic and non-diabetic patients

^a^Single institution study.

ACE = antiplatelet coagulation exactness; BMI = body mass index; CAD = coronary artery disease; DOAC = direct-acting oral anticoagulant; DVT = deep vein thrombosis; MALE = major adverse limb event; PAD = peripheral arterial disease; PE = pulmonary embolism; TEG = thromboelastography; TEG-PM = thromboelastography with platelet mapping

**Table 3:** Main study characteristics

Author (year)	Subtype of PAD	Anticoagulation regime	Antiplatelet regime	Open intervention	Endovascular intervention	Hybrid intervention	Bleeding prediction
Suarez Ferreira *et al* (2023)^20^	Lower limb*	Not applicable	Clopidogrel	18%	61%	20%	Not discussed
Hall *et al* (2022)^21^	Lower limb*	Mixture of heparin, warfarin and direct-acting oral anticoagulants	Aspirin or clopidogrel or DAPT	Not defined	Not defined	Not defined	Not discussed
Hall *et al* (2023)^22^	Lower limb*	Direct-acting oral anticoagulant	MAPT or DAPT	36%	50%	14%	Not discussed
Lee *et al* (2024)^23^	Lower limb*	Not applicable	MAPT initially to DAPT at subsequent visit	12%	59%	28%	2 bleeding events (1 immediate postoperatively and 1 at 3 months postoperatively)
Majumdar *et al* (2022)^24^	Lower limb*	Mixture of heparin, warfarin and direct-acting oral anticoagulants	Aspirin or clopidogrel or DAPT	35%	49%	17%	Not discussed
Martin *et al* (1994)^25^	Not defined	Heparin intravenous 5,000 IU intraoperatively	Not applicable	100%	0%	0%	Not discussed
Suarez *et al* (2024)^26^	Lower limb claudication 38% Critical limb-threatening ischaemia 58% Unknown 9%	Unspecified	Unspecified (subgroup: ACE using TEG-PM to maintain platelet inhibition >29%)	31%	45%	15%	No bleeding events (after implementing ACE protocol)
Suarez Ferreira *et al* (2024)^27^	Lower limb*	Not applicable	Aspirin monotherapy	41%	42%	17%	Not discussed
Suarez Ferreira *et al* (2024)^28^	Lower limb claudication 30% Critical limb-threatening ischaemia 45% Unknown 25%	Mixture of heparin and warfarin	Mixture of MAPT and DAPT	26%	59%	14%	Not discussed
Shankar *et al* (2006)^29^	Part 1: Lower limb claudication 50% Critical limb-threatening ischaemia 50% Part 2: Claudication 27% Critical limb-threatening ischaemia 73%	Not applicable	Aspirin monotherapy	Not applicable	Not applicable	Not applicable	Not applicable
Hall *et al* (2024)^30^	Lower limb*	Mixture of heparin, warfarin and direct-acting oral anticoagulant	Mixture of MAPT and DAPT	33%	48%	17%	Not discussed
Majumdar *et al* (2022)^31^	Lower limb*	Full-dose anticoagulation (not clearly defined)	Mixture of MAPT and DAPT	Not defined	Not defined	Not defined	Not discussed
Majumdar *et al* (2023)^32^	Lower limb*	Not applicable	Mixture of MAPT and DAPT	38%	44%	17%	Not discussed
Lee *et al* (2024)^33^	Lower limb*	Unspecified	Mixture of MAPT, DAPT, and anticoagulation	32%	49%	19%	Not discussed

*no characterisation of either claudication or critical limb-threatening ischaemia. ACE = antiplatelet coagulation exactness; DAPT = dual antiplatelet therapy; MAPT = mono antiplatelet therapy; TEG = thromboelastography; TEG-PM = thromboelastography with platelet mapping

**Table 4:** Quality assessment of non-randomised studies (Newcastle–Ottawa scale)

Author (year)	Representativeness of exposed cohort	Selection of the non-exposed cohort	Ascertainment of exposure	Demonstration that outcome of interest was not present at the start of the study	Comparability of cohorts	Assessment of outcome	Follow-up period	Follow-up adequacy	Total (of 9)
Suarez Ferreira *et al* (2023)^20^	★	★	★		★	★	★	★	7
Hall *et al* (2022)^21^	★	★	★	★	★★	★	★	★	9
Hall *et al* (2023)^22^	★	★	★	★	★★	★	★	★	9
Lee *et al* (2024)^23^	★	★	★	★	★★	★	★	★	9
Majumdar *et al* (2023)^24^	★	★	★	★	★★	★	★	★	9
Martin *et al* (1994)^25^	★		★		★	★			4
Suarez *et al* (2024)^26^	★	★	★	★	★★	★	★	★	9
Suarez Ferreira *et al* (2024)^27^	★	★	★	★	★★	★	★	★	9
Suaraez Ferreira *et al* (2024)^28^	★	★	★	★	★★	★	★	★	9
Shankar *et al* (2006)^29^	★		★		★	★	★		5
Hall *et al* (2024)^30^	★	★	★	★	★★	★	★	★	9
Majumdar *et al* (2022)^31^	★	★	★	★	★★	★	★	★	9
Majumdar *et al* (2023)^32^	★	★	★	★	★★	★	★	★	9
Lee *et al* (2024)^33^	★	★	★	★	★★	★	★	★	9

### Prediction of thrombotic events

Nine studies reported on the ability to predict thrombotic events. All the studies were based on TEG-PM. In three studies, patients experiencing major adverse limb events (MALE)/thrombotic events showed decreased platelet inhibition.^21,22,24^ More anticoagulation usage including DOAC was noted in those experiencing thrombotic events/MALE.^22,24^ In two studies, women had significantly more thrombotic events than men, despite being on the same medication in one study, thereby highlighting the sex based difference in response to antiplatelet therapy.^26,^^27^ Suarez Ferreira *et al* reported that increased clot strength (maximum amplitude [MA]) predicted thrombosis within 30 days.^28^ Hall *et al* reported that patients experiencing thrombotic events were more often diabetic and on full-dose anticoagulation.^30^ TEG-PM profiles in diabetic patients revealed increased MA. Lee *et al* reported that in diabetic patients, elevated haemoglobin A_1c_ (HbA_1c_) is associated with prothrombotic tendency: diabetic patients with HbA_1c_ >6.5% at baseline had significantly higher MA.^33^ Lee *et al* also reported increased MA in diabetic patients using the functional fibrinogen assay (citrated functional fibrinogen assay) at 1 month post intervention, highlighting the contribution of fibrinogen to clot strength. Lee *et al* reported that platelet reactivity is higher in endovascular interventions and transitioning to dual antiplatelet therapy (DAPT) reduced platelet aggregation. The results are illustrated in Table 5.^23^

**Table 5:** Ability to predict thrombosis

Author (year)	TEG/TEG-PM	Outcome studied	Significant parameter in TEG/TEG-PM	Antiplatelet regime	Anticoagulation regime	Result
Hall *et al* (2022)^21^	TEG-PM	Identify the utility of viscoelastic assays in defining the coagulation profiles of complex cardiovascular patients	ADP–platelet aggregation and platelet inhibition, and MA	Significantly lower samples on DAPT, as well as clopidogrel monotherapy in CAD and PAD group	Significantly more samples on DOAC in PAD and CAD cohort. Significantly more samples on full-dose anticoagulation in CAD and PAD cohort	Increased ADP–platelet aggregation and decreased platelet inhibition in patients with CAD and PAD, as well as in patients with MALE
Hall *et al* (2023)^22^	TEG and TEG-PM	• Compare TEG-PM metrics between DOAC vs non-DOAC • Incidence of MALE, the associated antithrombotic regimen and TEG-PM profile	R-time ADP and AA platelet aggregation and platelet inhibition	Non-DOAC cohort: 30% DAPT 68% MAPT	DOAC cohort (27%): 21% DOAC + DAPT 66% DOAC + MAPT 13% DOAC only	Prolonged R-time, decreased platelet inhibition in DOAC Patients experiencing MALE are more often on DOAC and demonstrate prolonged R-time and increased clot strength MA
Lee *et al* (2024)^23^	TEG-PM	• Compare clinical outcomes (occurrence of by-pass/in-stent stenosis; wound complications; bleeding events) in patients who transitioned from MAPT to DAPT • Influence of sex on efficacy of MAPT and DAPT • Compare platelet reactivity between open and endovascular procedures to elucidate the impact of the procedure on response to DAPT • Longitudinal comparison of antiplatelet reactivity	Platelet inhibition (ADP% and AA%) Platelet aggregation (ADP% and AA%)	81% on aspirin MAPT and then transitioned to DAPT 19% on clopidogrel MAPT and then transitioned to DAPT	No DOAC patients	• No statistically significant difference in occurrence of clinical outcomes • MAPT and DAPT are comparably effective across both sexes • Endovascular patients have high platelet reactivity and transitioning to DAPT reduces aggregation • Both aspirin and clopidogrel MAPT patients showed significant reduction in AA% platelet inhibition (100% for clopidogrel vs 34.6% for aspirin) when transitioned to DAPT; significant reduction in ADP% platelet inhibition in aspirin MAPT patients on transitioning to DAPT
Majumdar *et al* (2023)^24^	TEG-PM	Comparison of TEG-PM metrics between event and non-event groups	R-time % Platelet aggregation % Platelet inhibition	84.6% on antiplatelet therapy (63.6% on MAPT and 21% on DAPT)	Significantly greater use of anticoagulation in thrombosis group (56.7% vs 33.3%) including the use of DOAC (40% vs 18.2%)	Patients with thrombosis had greater platelet aggregation and low platelet inhibition. For every 1% increase in platelet aggregation, the hazard of experiencing an event is increased by 5%
Suarez *et al* (2024)^26^	TEG and TEG-PM	Measure the impact of sex on platelet function and response to antiplatelet therapy	MA and % platelet inhibition	Not clear ACE algorithm (only for 34 patients as a pilot): Patients are started on DAPT post re-vascularisation and platelet inhibition measured with TEG-PM 7 days post treatment. If platelet inhibition is <29%, clopidogrel is replaced with ticagrelor	Not clear	• Significantly higher thrombotic rates in women than men taking the same medication • Women taking aspirin showed greater clot strength and decreased platelet inhibition compared with men
Suarez Ferreira *et al* (2024)^27^	TEG-PM	Delineate the difference in response to aspirin monotherapy between women and men	MA and platelet inhibition	Aspirin monotherapy (100%)	Not on anticoagulants	Women showed higher rate of occlusion at the area of intervention
Suarez Ferreira *et al* (2024)^28^	TEG-PM	Determine whether objective measures of clot strength predict high risk of thrombosis	MA (ADP, thrombin and fibrin separately)	No difference in usage of MAPT and DAPT between thrombosis and no thrombosis groups	No difference in usage of DOAC, and warfarin between thrombosis and no thrombosis groups	• Significantly greater MA (ADP, thrombin and fibrin) in thrombosis cohort • Increased clot strength is predictive of thrombosis/stenosis within 30 days
Hall *et al* (2024)^30^	TEG-PM	Thrombosis risk stratification based on metabolic comorbidities using TEG-PM	Platelet aggregation, platelet inhibition, MA (ADP, and AA)	No difference in usage of MAPT and DAPT between diabetic and non-diabetic patients; and no also between BMI<25 and BMI>25 groups	Significantly higher use of anticoagulation in diabetic patients, no significant difference in use of DOAC between the two groups. No difference in usage of anticoagulation and DOAC between BMI<25 and BMI>25 groups	• Significantly increased reaction time, and MA (ADP and AA) in diabetic patients • Significantly higher MA (ADP) and platelet aggregation and significantly lower platelet inhibition in BMI>25 group • Patients with thrombosis were more often on full-dose anticoagulation and were diabetic
Lee *et al* (2024)^34^	TEG-PM	Identify the association between different coagulation parameters and variations in HbA_1c_	MA ADP and AA Functional fibrinogen assay-Activator F MA, CFF MA	Significantly more participants in the diabetes cohort on DAPT at baseline No difference in antiplatelet therapy usage between thrombosis and no thrombosis groups	No difference in anticoagulant therapy usage between diabetes and no diabetes groups No difference in anticoagulant therapy usage between thrombosis and no thrombosis groups	Elevated HbA1c appears to be associated with prothrombotic tendency • Significantly increased ADP MA, AA MA, Act F MA and heparinase MA in diabetic patients with HbA_1c_>6.5 at baseline • Significantly increased CFF MA in diabetic patients with HbA_1c_>6.5 at 1 month postoperatively

AA = arachidonic acid; ACE = antiplatelet coagulation exactness; ActF = Activator F; ADP = adenosine diphosphate; BMI = body mass index; CAD = coronary artery disease; DAPT = dual antiplatelet therapy; DOAC = direct-acting oral anticoagulant; MA = maximum amplitude; MALE = major adverse limb event; MAPT = mono antiplatelet therapy; PAD = peripheral arterial disease; R-time = reaction time; TEG = thromboelastography; TEG-PM = thromboelastography with platelet mapping

### Utility of TEG and use for personalised anticoagulation

The results of TEG utility and use for personalised anticoagulation are depicted in Table 6. Overall, four studies highlighted the use of TEG-PM for personalising thromboprophylaxis.^20,26,27,32^ Suarez Ferreira *et al* reported that an alternative antiplatelet other than clopidogrel is needed in patients taking atorvastatin to achieve optimal thromboprophylaxis.^20^ Suarez *et al* reported that after implementation of the antiplatelet coagulation exactness (ACE) algorithm, antiplatelet therapy needed to be personalised based on response, and noted a significant decrease in thrombotic events without increased bleeding risk. Suarez Ferreira *et al* reported that women need a higher dose of aspirin and or additional antiplatelet therapy to achieve adequate thromboprophylaxis.^26,^^27^ Majumder *et al* reported the difference in response to different antiplatelet agents at different time points in the clinical course, thereby highlighting the importance of personalising treatment to achieve optimal outcomes.^32^

**Table 6:** Other utility and use to personalise anticoagulation.

Author (year)	TEG/ TEG-PM utility	TEG/TEG-PM parameter	Result	Conclusion
Suarez Ferreira *et al* (2023)^20^	Delineate the difference in platelet function between patients on clopidogrel alone vs clopidogrel and atorvastatin undergoing peripheral re-vascularisation	Platelet inhibition	Overall greater platelet inhibition in clopidogrel alone	Alternative antiplatelet other than clopidogrel is needed to achieve adequate thromboprophylaxis in patients taking atorvastatin
Martin *et al* (1994)^25^	Intraoperative TEG monitoring may assist in providing steady-state anticoagulation	TEG coagulation profile and ACT	Significant decline in ACT values at 30 and 60min post heparinisation. TEG profile abolished immediately after heparin administration. Near complete return of TEG coagulation profile in 2 patients prior to completion of procedure with corresponding ACT values <160s	Variability of patient response to heparinisation necessitates intraoperative monitoring of anticoagulation
Suarez *et al* (2024)^26^	TEG-PM metrics can be utilised to personalise antiplatelet therapy based on individual response	ADP–platelet aggregation and ADP–platelet inhibition	Significant decrease in thrombosis after implementation of ACE algorithm without increasing bleeding events	TEG-PM may mitigate the sex-specific outcome disparities caused by inadequate thromboprophylaxis
Suarez Ferreira *et al* (2024)^27^	TEG-PM highlight the difference between men and women in antiplatelet response to aspirin monotherapy	MA and platelet inhibition	Significantly greater clot strength (MA) and less platelet inhibition in women compared with men	TEG-PM metrics highlight that women need higher doses of aspirin, and/or additional antiplatelet medication to achieve the same therapeutic effect as men
Shankar *et al* (2006)^29^	TEG can show the effect of contrast agent on coagulation at the local site in a real-life setting (blood mixed with contrast as compared to blood alone)	R-time, K-time, angle, MA, CI	Significant increase in R-time and K-time; significant decrease in angle, MA and CI after contrast injection	Iohexol reduces blood coagulability during diagnostic angiography
Majumdar *et al* (2022)^31^	TEG-PM assay identifies patients at high risk for infection and underlines the importance of increased observation, and/or enhanced antimicrobial/antithrombotic therapy in this high-risk group	Platelet aggregation, platelet inhibition, and MA	• Incidence of surgical site infection/dehiscence was 18.6% • Significantly higher platelet aggregation and MA in the event group • Significantly reduced platelet inhibition in the event group • Platelet aggregation is an independent and consistent predictor of infection (HR = 1.04, 95% confidence interval 1.03–1.06; *p* < 0.01)	Based on cut point analysis, cut-points of >33.2 MA, >46.6% platelet aggregation, or <55.8% platelet inhibition identifies patients at high risk of infection
Majumdar *et al* (2023)^32^	TEG-PM highlights the difference in response to different antiplatelet therapy and also the difference in response during different phases of clinical course	Platelet aggregation, and platelet inhibition	• Higher platelet inhibition with clopidogrel 75mg compared with those on either dose of aspirin (81mg or 325mg) • Higher platelet inhibition and less platelet aggregation with samples on DAPT in the preoperative phase, and postoperative outpatient phase. No significant difference in the postoperative inpatient phase	Significant variability in response to both MAPT and DAPT

ACE = antiplatelet coagulation exactness; ACT = activated coagulation time; ADP = adenosine diphosphate; CI = coagulation index; DAPT = dual antiplatelet therapy; HR = hazard ratio; K-time = kinetic time; MA = maximum amplitude; R-time = reaction time; TEG = thromboelastography; TEG-PM = thromboelastography with platelet mapping

Three studies reported the other utility of TEG.^25,29,31^ Martin *et al* reported that intraoperative monitoring of coagulation with TEG is needed to achieve steady-state anticoagulation.^25^ Shankar *et al* reported coagulation parameters using TEG at the site of ionic contrast injection aortic blood samples and reflected on endogenous heparin activity.^29^ Ionic contrast agent use during diagnostic angiographic studies does not increase blood coagulability. Majumdar *et al* reported that TEG-PM parameters (MA, platelet aggregation and platelet inhibition) can identify patients at high risk of infections.^31^

## Discussion

Antiplatelet therapy and anticoagulants are integral elements in managing PAD and thus adequate monitoring and implementation of these therapies are vital. This review identified potential applications of TEG in PAD, more specifically TEG-PM in predicting outcomes and tailoring thromboprophylaxis.

### Utility of TEG and TEG-PM in predicting outcomes in PAD

Of the included studies, many factors were found to influence TEG-PM parameters in PAD patients to favour thrombosis. These included: increasing HbA_1c_ (increase in ADP MA); diabetes (increase MA, both AA and ADP and increased reaction (R)-time); female sex (increased MA Activator F [ActF] and MA AA, and lower platelet inhibition compared with males); increasing body mass index (BMI) >25 (lower ADP–platelet inhibition and increased ADP–platelet aggregation compared with PAD patients with normal BMI); and use of atorvastatin along with clopidogrel (reduced platelet inhibition), thereby demonstrating potential utility of TEG-PM in personalising thromboprophylaxis in these patient groups.^20,^^26,^^27,^^30,^^33^

Hall *et al* investigated the role of TEG-PM in predicting outcomes for patients with PAD and simultaneous significant coronary artery disease (CAD) undergoing lower limb re-vascularisation.^21^ Approximately 30% of the study population had PAD and co-existing CAD and this group had a higher incidence of atrial fibrillation. Thus, the PAD with CAD group were more likely to be on full-dose anticoagulation as opposed to being on clopidogrel or dual antiplatelets. Interestingly, the study demonstrated that in the PAD with CAD group there were altered TEG-PM profiles (increased platelet reactivity–lower platelet inhibition, higher platelet aggregation) and higher MA profiles. The lower platelet inhibition noted is likely due to the increased systemic anticoagulation and lack of antiplatelet therapy in this group. The altered TEG-PM profiles in this study were found to be associated with an increased risk of MALEs. It was also noted that MALEs were more common in the PAD with significant CAD group than the PAD alone group, possibly for the same reason of lower platelet inhibition in the former. Furthermore, patients who experienced MALEs had greater ADP–platelet aggregation and decreased ADP–platelet inhibition, preceding the adverse event by an average of 40.8 days. One challenge encountered in this study is the heterogeneity of the patient population studied.

In a different study on patients undergoing lower limb re-vascularisation, Hall *et al* divided the patients into five groups: DOAC only; DOAC and concomitant DAPT; DOAC and concomitant mono antiplatelet therapy (MAPT); DAPT only; and MAPT only.^22^ Interestingly, via TEG-PM, the DOAC and MAPT group compared with MAPT alone demonstrated a longer time to clot formation (R-time) and less platelet inhibition, whereas patients on DAPT demonstrated significantly greater platelet inhibition compared with both the DOAC and MAPT group and the MAPT alone group. Patients who experienced MALE were more likely to be on DOAC therapy and also demonstrated a significantly longer R-time and increased MA. These results indicate that it might be possible to optimise treatment approaches to predict and prevent adverse outcomes using platelet mapping in patients undergoing lower limb re-vascularisation who are anticoagulated. Hall *et al* also dispute the ‘blanket approach’ to antiplatelet and anticoagulant therapy in PAD. The challenge in this patient group would be balancing both thrombotic and bleeding risk when considering the addition of a further antiplatelet, and TEG-PM could facilitate this.

When predicting thrombosis post re-vascularisation, Suarez Ferreira *et al* established that an increase in MA was found to be predictive of thrombosis/stenosis within 30 days.^28^ In the study, 18% of PAD patients who underwent re-vascularisation experienced thrombosis. The thrombosis cohort exhibited significantly greater MA ADP, MA fibrin and MA thrombin. In a different study of 162 patients following re-vascularisation, 18.5% experienced graft/stent thrombosis and had significantly greater platelet aggregation; Cox proportional hazards regression analysis revealed that for every 1% increase in platelet aggregation, the hazard of experiencing an event during the study period increased by 5%.^24^

In another study of PAD patients undergoing lower limb re-vascularisation, Majumdar *et al* demonstrated that higher MA, higher platelet aggregation and lower platelet inhibition are predictors of postoperative wound infection.^31^ On average, the results were derived from samples preceding the event by 19.5 days. Of the 102 patients enrolled, 19 developed infection wound/dehiscence, graft infection or limb amputation because of uncontrolled infection. One-third of the event group also had a concomitant thrombosis. It is therefore suggested that TEG could be implemented to assess both thrombotic and infection risk, thereby significantly improving patient outcomes**.**

Martin *et al* highlighted the benefit of using TEG to monitor intraoperative anticoagulation and its superiority to activated coagulation time (ACT), whereas Shankar *et al* using TEG have demonstrated that intraoperative blood coagulability is reduced by ionic contrast agent injection during diagnostic angiographic studies.^25,^^29^

The reviewed studies suggest that TEG-PM could be a valuable tool in predicting outcomes in patients with PAD and guide tailored antiplatelet therapy to ensure optimum therapeutic efficacy by balancing thrombotic against bleeding risk. It is important to acknowledge that this could merely be an association, and whether tailoring thromboprophylaxis based on TEG-PM leads to improved outcomes is yet to be proved.

### Potential use and limitations of TEG in clinical practice

Conventional coagulation tests were developed principally to monitor anticoagulants and are less suitable for assessing thrombotic risk. These tests analyse platelet-poor plasma and only capture the time to the start of clot formation, as opposed to TEG, which analyses a whole blood sample and captures all stages of clot formation, maximum clot strength and clot lysis.^1,2,10^ As identified in studies discussed above, many patients with PAD may be commenced on anticoagulant therapy and still may be at risk of thrombosis due to an absence of assessment of platelet function using conventional coagulation tests. Furthermore, many patient factors are frequently observed in PAD such as diabetes, obesity, female sex and significant cardiac disease that can influence clot dynamics, and their impact is not accounted for via conventional coagulation tests. Varying responses to antiplatelet therapy have been observed in PAD, including resistance.^32,^^34^ Current evidence does not support the use of standard laboratory tests in guiding antiplatelet therapy in patients with resistance and there is no set standard for monitoring antiplatelet therapy.^12,^^35^ Thus, there is large scope for implementation of TEG-PM in PAD.

Despite its advantages, TEG does have limitations. From an operator perspective, obtaining reliable and reproducible results may not be achieved by inadequately trained operators or by using different equipment.^1,36^ Unlike standard coagulation tests, samples analysed via TEG do not undergo robust laboratory quality-control processes.^37^ This can be further complicated by variability in TEG technique (addition of different activators and modifications). Standardisation of results is also required, and baseline TEG measurements should be obtained for use as a comparator. In most of the included studies baseline (pre-intervention) TEG measurements were available as a comparator. In addition, there are no set cut-off values to act upon to guide treatment. However, steps have already been made to mitigate some of these limitations, such as the introduction of software to standardise results and standardisation of the activators used.^36^ In addition, a majority of the studies included in our review originated from the same centre, and hence our results should be interpreted with caution. The exclusion of studies not in English, as well as publication bias are additional factors to be considered. Furthermore, there was significant heterogeneity (patient profiles, different TEG parameters analysed, variation in timings of measurement and antithrombotic regimens) in the included studies that precluded a subgroup analysis. The only recurrent theme in most of the studies was that a greater proportion of patients underwent endovascular intervention. The significance of this is unknown. Despite these, the majority of the included studies were of high quality (as per the NOS) with the exclusion of only two low-quality studies, one of which did not analyse the impact of TEG on interventions, thereby excluding any potential bias based on the quality of the included studies.^29^

### Practical implications

Considerations regarding the feasibility of TEG should include availability, cost and training requirements. Although TEG is widely used in clinical practice, ascertaining its availability in various clinical settings is beyond the scope of our review. TEG and TEG-PM require trained personal to perform and interpret the findings. Machine cost and associated expenses, including service contracts, consumables, quality-control materials and cost for training clinical personnel, must be weighed against the potential cost reductions associated with theatre time, readmissions, length of hospital stay, conventional tests, complications (bleeding and thrombotic) and reinterventions. There is a need for high-quality data defining cost-effectiveness in this particular clinical setting to guide financial viability.

### Future research directions

There are crucial research gaps to be addressed, particularly the optimal timing of TEG/TEG-PM in the post-intervention phase, the value of TEG over other conventional tests and any cost–benefit advantage. To address this, well-designed randomised control trials and multicentric studies are needed.

## Conclusions

This systematic review highlights the valuable applications of TEG-PM in PAD, in predicting thrombotic risk, and its use in personalising thromboprophylaxis. TEG-PM could potentially improve the outcomes in PAD further; however, robust evidence is needed to influence practice change.

## Conflicts of interest

The authors declare no conflicts of interest.

## Funding

This research received no external funding.

## Author contributions

Conceptualisation, JPG. Methodology, JPG and AA. Data collection and screening, JAB and JPG. Validation, JAB and JPG. Quality of studies assessment, JAB and JPG. AA solved any disagreement in data screening and quality assessment. Formal analysis, JPG and JAB. Writing—original draft preparation, JAB and JPG. Writing—review and editing, JAB, JPG and AA. Supervision, JPG and AA. Project administration, JAB. All authors have read and agreed to the final version of the manuscript.

## Artificial Intelligence

The author/s declare that no AI was used to conduct the study or prepare the manuscript.

## Institutional review board statement

This study did not require ethical approval.

## Informed consent statement

Patient consent was not needed as is not applicable due to the study being a review of published studies.

## Data availability

Data for this study were synthesised from publicly available databases (PubMed and Embase).

## References

[1] Shaydakov ME, Sigmon DF, Blebea J. Thromboelastography [Updated 2023 Apr 10]. In: StatPearls [Internet]. Treasure Island (FL): StatPearls Publishing; 2024. https://www.ncbi.nlm.nih.gov/sites/books/NBK537061/30725746

[2] Selby R. “TEG talk”: expanding clinical roles for thromboelastography and rotational thromboelastometry. *Hematology Am Soc Hematol Educ Program* 2020; **1**: 67–75. 10.1182/hematology.2020000090 PMC7727516 33275705

[3] Cohen T, Haas T, Cushing MM. The strengths and weaknesses of viscoelastic testing compared to traditional coagulation testing. *Transfusion* 2020; **60**: S21–S28. 33089934 10.1111/trf.16073

[4] Kankaria A, Majumdar M, Lee S *et al.* Platelet function testing and clinical outcomes in peripheral arterial disease: systematic review and narrative synthesis. *J Vasc Surg* 2024; **80**: 269–278. 38122860 10.1016/j.jvs.2023.12.028

[5] Whitton TP, Healy WJ. Review of thromboelastography (TEG): medical and surgical applications. *Ther Adv Pulm Crit Care Med* 2023; **18**: 29768675231208426. 38107072 10.1177/29768675231208426 PMC10725099

[6] Kim Y, Patel SS, McElroy IE *et al.* A systematic review of thromboelastography utilization in vascular and endovascular surgery. *J Vasc Surg* 2022; **75**: 1107–1115. 34788649 10.1016/j.jvs.2021.11.037

[7] Yang JK, Jimenez JC, Jabori S. Antiplatelet therapy before, during, and after extremity revascularization. *J Vasc Surg* 2014; **60**: 1085–1091. 25124360 10.1016/j.jvs.2014.07.006

[8] Canonico ME, Piccolo R, Avvedimento M *et al.* Antithrombotic therapy in peripheral artery disease: current evidence and future directions. *J Cardiovasc Dev Dis* 2023; **10**: 164. 37103043 10.3390/jcdd10040164 PMC10144744

[9] Society for Vascular Surgery Lower Extremity Guidelines Writing Group; Conte MS, Pomposelli FB *et al*. Society for Vascular Surgery practice guidelines for atherosclerotic occlusive disease of the lower extremities: management of asymptomatic disease and claudication. J Vasc Surg 2015; **61**(3 Suppl): 2S–41S. Epub 2015 Jan 28. Erratum in: J Vasc Surg 2015; 61(5): 1382. PMID: 25638515. 25638515 10.1016/j.jvs.2014.12.009

[10] Lim HY, O'Malley C, Donnan G *et al.* A review of global coagulation assays — Is there a role in thrombosis risk prediction? *Thromb Res* 2019; **179**: 45–55. 31078120 10.1016/j.thromres.2019.04.033

[11] Amaraneni A, Chippa V, Goldin J *et al.* Anticoagulation Safety. [Updated 2024 Oct 6]. In: StatPearls [Internet]. Treasure Island (FL): StatPearls Publishing; 2024. https://www.ncbi.nlm.nih.gov/books/NBK519025/30085567

[12] Davidson S. Monitoring of antiplatelet therapy. *Methods Mol Biol* 2023; **2663**: 381–402. 37204725 10.1007/978-1-0716-3175-1_25

[13] Angiolillo DJ, Fernandez-Ortiz A, Bernardo E *et al.* Variability in individual responsiveness to clopidogrel: clinical implications, management, and future perspectives. *J Am Coll Cardiol* 2007; **49**: 1505–1516. 17418288 10.1016/j.jacc.2006.11.044

[14] Serebruany VL, Steinhubl SR, Berger PB, *et al.* Variability in platelet responsiveness to clopidogrel among 544 individuals. *J Am Coll Cardiol* 2005; **45**: 246–251. 15653023 10.1016/j.jacc.2004.09.067

[15] Gasecka A, Zimodro JM, Appelman Y. Sex differences in antiplatelet therapy: state-of-the art. *Platelets* 2023; **34**: 2176173. 36809993 10.1080/09537104.2023.2176173

[16] Eikelboom JW, Hirsh J, Weitz JI *et al.* Aspirin-resistant thromboxane biosynthesis and the risk of myocardial infarction, stroke, or cardiovascular death in patients at high risk for cardiovascular events. *Circulation* 2002; **105**: 1650–1655. 11940542 10.1161/01.cir.0000013777.21160.07

[17] Gurbel PA, Bliden KP, Hiatt BL, O'Connor CM. Clopidogrel for coronary stenting: response variability, drug resistance, and the effect of pretreatment platelet reactivity. *Circulation* 2003; **107**: 2908–2913. 12796140 10.1161/01.CIR.0000072771.11429.83

[18] Page MJ, McKenzie JE, Bossuyt PM *et al.* The PRISMA 2020 statement: an updated guideline for reporting systematic reviews. *BMJ* 2021; **372**: n71. 33782057 10.1136/bmj.n71 PMC8005924

[19] Howick J, Chalmers I, Glasziou P. *Explanation of the 2011 Oxford Centre for Evidence-Based Medicine (OCEBM) Levels of Evidence (Background Document)*. Oxford: Oxford Centre for Evidence-Based Medicine; 2011.

[20] Suarez Ferreira SP, Hall RP, Majumdar M *et al.* Atorvastatin effect on clopidogrel efficacy in patients with peripheral artery disease. *Ann Vasc Surg* 2023; **95**: 74–79. 37257642 10.1016/j.avsg.2023.05.023 PMC10524645

[21] Hall R, Majumdar M, Cassidy R *et al.* Use of thromboelastography with platelet mapping to identify prothrombotic coagulation profiles in patients with history of cardiac intervention undergoing lower extremity revascularization. *J Am Coll Surg* 2023; **236**: 495–504. 36729802 10.1097/XCS.0000000000000497

[22] Hall RP, Majumdar M, Ferreira SS *et al.* Impact of factor Xa inhibition on coagulation, platelet reactivity, and thrombosis in patients with peripheral artery disease. *Ann Vasc Surg* 2023; **97**: 211–220. 37657677 10.1016/j.avsg.2023.08.004

[23] Lee I, Suarez S, Hall R, *et al.* Optimizing platelet inhibition in peripheral artery disease: A comparison of mono-antiplatelet therapy and dual-antiplatelet therapy using thromboelastography. *Vascular* 2024; **33**: 3–18. 38441042 10.1177/17085381241237005

[24] Majumdar M, Hall RP, Feldman Z *et al.* Predicting arterial thrombotic events following peripheral revascularization using objective viscoelastic data. *J Am Heart Assoc* 2023; **12**: e027790. 36565191 10.1161/JAHA.122.027790 PMC9973575

[25] Martin P, Greenstein D, Gupta NK *et al.* Systemic heparinization during peripheral vascular surgery: thromboelastographic, activated coagulation time, and heparin titration monitoring. *J Cardiothorac Vasc Anesth* 1994; **8**: 150–152. 8204807 10.1016/1053-0770(94)90053-1

[26] Suarez S, Agrawal A, Patel S *et al.* The impact of sex on antiplatelet and anticoagulant thromboprophylaxis in patients with peripheral artery disease post-revascularization. *Ann Surg* 2024; **280**: 463–472.38860382 10.1097/SLA.0000000000006375

[27] Suarez Ferreira SP, Hall RP, Morrow K *et al.* The impact of sex on platelet responses to aspirin in patients with peripheral artery disease. *Am J Hematol* 2024; **99**: S6–S12. 38400527 10.1002/ajh.27258 PMC11023782

[28] Suarez Ferreira S, Agrawal A, Lee I *et al.* The use of clot strength as a predictor of thrombosis in peripheral artery disease. *Ann Vasc Surg* 2024; **109**: 273–283. 39069123 10.1016/j.avsg.2024.06.041 PMC11524772

[29] Shankar VK, Handa A, Philips-Hughes J *et al.* Thromboelastographic changes following nonionic contrast medium injection during transfemoral angiography in patients with peripheral arterial occlusive disease. *Cardiovasc Intervent Radiol* 2006; **29**: 1046–1052. 16810462 10.1007/s00270-004-0207-1

[30] Hall R, Suarez S, Majumdar M *et al.* Thromboelastography with platelet mapping identifies high platelet reactivity is associated with obesity, diabetes, and thrombotic events. *Ann Vasc Surg* 2024; **104**: 227–236. 38490537 10.1016/j.avsg.2023.12.079

[31] Majumdar M, Lella S, Hall RP *et al.* Utilization of thromboelastography with platelet mapping to predict infection and poor wound healing in postoperative vascular patients. *Ann Vasc Surg* 2022; **87**: 213–224. 35339591 10.1016/j.avsg.2022.03.008

[32] Majumdar M, Waller D, Poyant J *et al.* Variability of antiplatelet response in patients with peripheral artery disease. *J Vasc Surg* 2023; **77**: 208–215.e3. 36028157 10.1016/j.jvs.2022.08.015

[33] Lee I, Agrawal A, Ghandour S *et al.* The influence of diabetes on thrombotic profiles and outcomes on patients with peripheral artery disease. *Ann Vasc Surg* 2024; 110: 246–259. 39067844 10.1016/j.avsg.2024.06.035

[34] Guirgis M, Thompson P, Jansen S. Review of aspirin and clopidogrel resistance in peripheral arterial disease. *J Vasc Surg* 2017; **66**: 1576–1586. 28893489 10.1016/j.jvs.2017.07.065

[35] Michelson AD, Deepak L. Bhatt; How I use laboratory monitoring of antiplatelet therapy. *Blood* 2017; **130**: 713–721. 28600334 10.1182/blood-2017-03-742338

[36] Korpallová B, Samoš M, Bolek T *et al.* Role of thromboelastography and rotational thromboelastometry in the management of cardiovascular diseases. *Clin Appl Thromb Hemost* 2018; **24**: 1199–1207. 30041546 10.1177/1076029618790092 PMC6714776

[37] Kitchen DP, Kitchen S, Jennings I *et al.* Quality assurance and quality control of thrombelastography and rotational thromboelastometry: the UK NEQAS for blood coagulation experience. *Semin Thromb Hemost* 2010; **36**: 757–763. 20978996 10.1055/s-0030-1265292

